# Stages of behavioural change after direct-to-consumer disease risk profiling: study protocol of two integrated controlled pragmatic trials

**DOI:** 10.1186/s13063-018-2630-7

**Published:** 2018-04-19

**Authors:** Kelly F. J. Stewart, Anke Wesselius, Annemie M. W. J. Schols, Maurice P. Zeegers

**Affiliations:** 1 Department of Complex Genetics, NUTRIM School of Nutrition and Translational Research in Metabolism, Maastricht University, Maastricht, The Netherlands; 2 Department of Respiratory Medicine, NUTRIM School of Nutrition and Translational Research in Metabolism, Maastricht University, Maastricht, The Netherlands; 3 CAPHRI School for Public Health and Primary Care, Maastricht University, Maastricht, The Netherlands

**Keywords:** Personalised medicine, Personalised prevention, Trials, Direct-to-consumer genetics, Lifestyle epidemiology, Genetic epidemiology, Community genetics, Health promotion

## Abstract

**Background:**

The incidence and prevalence of chronic diseases have reached epidemic proportions during the last decades and are not expected to diminish. Chronic diseases increasingly affect younger individuals too, with over 40% of all deaths due to non-communicable diseases occurring before the age of 70. This has led to the development of information services aimed at preventive health care, such as *Health Potential®*. This counselling service estimates a personal disease risk of a carefully selected list of preventable common chronic diseases that have both a genetic and a lifestyle component of development. The results are delivered face-to-face by a lifestyle counsellor, simultaneously stimulating initial steps towards behaviour change. This information can assist in lifestyle decision-making.

**Methods/design:**

The primary aim is to study the effect of the Health Potential® service on change in lifestyle behaviour in distinguishable customer populations. The secondary aims are (1) to study the effect of the Health Potential® service on determinants of behaviour change, (2) to study the effect of additional lifestyle counselling on behaviour change and determinants thereof, and (3) to describe the characteristics of the Health Potential® customer. The study consists of two integrated designs: (A) a two-armed non-randomised controlled pre-test/post-test trial (1.5:1 ratio), followed by (B) a two-armed randomised controlled pre-test/post-test trial (1:1 ratio), resulting in three study arms. Participants are clients of local prevention clinics, purchasing a personalised health check (PHC; intervention condition), consisting of Health Potential® and a general health check, or the general health check alone (GHC; control condition) (part A). PHC participants will be randomised to receive four additional lifestyle counselling sessions over a period of 3 months (part B).

**Discussion:**

This research can provide valuable insights into the effectiveness of and possible ways forward in the field of personalised prevention making use of lifestyle interventions enriched with modern genetic advancements.

**Trial registration:**

Nederlands Trial Register, NTR6289 and NTR6288. Registered on 24 February 2017.

**Electronic supplementary material:**

The online version of this article (10.1186/s13063-018-2630-7) contains supplementary material, which is available to authorized users.

## Background

The incidence and prevalence of chronic diseases have reached epidemic proportions during the last decades and are not expected to decrease or diminish. In 2012, 38 million people died as a result of a non-communicable disease, of which 82% were due to cardiovascular diseases, diabetes, cancer and chronic respiratory diseases [1]. This number is projected to increase to 52 million deaths by 2030 [2]. Of particular concern is the fact that over 40% of all NCD deaths occurred before the age of 70 in 2012 [1]. As a result of these developments, we must expect an increasing economic burden and reduction of quality of life in many societies, among both affected people and their social environment.

Most, if not all, common chronic diseases develop over a lifetime as a result of interplay between genetic predisposition and the accumulation of lifetime exposures to environmental and behavioural factors. According to the World Health Organization (WHO), unhealthy diet, physical inactivity, tobacco use and harmful alcohol consumption are among the most important modifiable causes of chronic disease worldwide [3]. For example, harmful alcohol use and tobacco use account for 12% of all deaths worldwide, and an additional 19% is accounted for by unhealthy diet and physical inactivity [3]. As these are all modifiable, great potential exists for prevention or delay of chronic diseases, resulting in a renewed necessity for and interest in disease prediction and prevention [4].

The fact that most chronic diseases seem to be preventable and the neoliberal political climate in many countries have contributed to the rise of personal responsibility for health [5]. Individuals are more and more expected to strive to obtain and maintain their best possible health. In order to do so, they need to be optimally informed and make their own risk-benefit analyses. This has led to an exponential growth in services aiming at assisting in informing and assisting individuals to make informed decisions. Another such approach is through genetic testing [6]. Although one has no control over their genetic make-up, knowledge of genetic predispositions could provide the opportunity for personalised prevention strategies and may serve in health-related decision-making [7, 8].

Personal genomic testing (PGT) uses genetic variations in individuals to estimate personal disease risks and sometimes several other genetically determined phenotypes such as food intolerances or drug metabolism [9]. PGT has become popular and accessible as a result of rapid advances in the field of DNA analysis techniques. This has made genome analysis possible at relatively low costs, with PGT companies like 23andMe dropping their price from US$999 in 2007 to US$199 in 2017 ([10], https://www.23andme.com/). These services are mostly offered to the general population direct-to-consumer (DTC), meaning without interference of a physician. PGT may help individuals change their behaviour towards more health-promoting lifestyle through identification of an increased disease risk [11]. An important assumption of the success of this strategy is that the identification of increased disease risk could directly influence risk reduction behaviours [8, 12, 13]. However, research has shown that this assumption mostly does not apply in all populations or that the effects are minor in general populations [13, 14].

Limitations of this idea that PGT may facilitate behaviour change are that, firstly, probability and genetic data are difficult to comprehend without counselling [15, 16] and understanding is strongly influenced by several factors such as the format of delivery [17]. Secondly, disease risks are not solely determined by either lifestyle or genetic factors, but both [18]. Thirdly, disease risk information alone may not be motivating enough for individuals to change behaviour and therefore additional lifestyle intervention and motivational counselling may be necessary to help individuals change and maintain behaviour [7].

To address these shortcomings, a new disease risk information service has been developed: *Health Potential®*. This service estimates a personal disease risk of a carefully selected list of preventable common chronic diseases that have both a genetic and a lifestyle component of development. The results are delivered face-to-face by a certified lifestyle counsellor, simultaneously stimulating initial steps towards behaviour change. Because previous research has shown that not all health care professionals feel confident in interpreting and communicating DTC-GT results to their patients or clients [19, 20], all Health Potential® counsellors are trained to ensure confidence and skill.

The effect of this new personalised service will be researched in two integrated studies, one observational study and one intervention trial, comparing the service to a general health check only. The primary aim of the study is to study the effect of the Health Potential® advice on change in lifestyle behaviour in distinguishable customer populations. The secondary aims are (1) to study the effect of the Health Potential® service on determinants of behaviour change, (2) to study the effect of additional lifestyle counselling on behaviour change and determinants thereof, and (3) to study the characteristics of the Health Potential® customer.

## Methods/design

### Study design

**Fig. 1:**
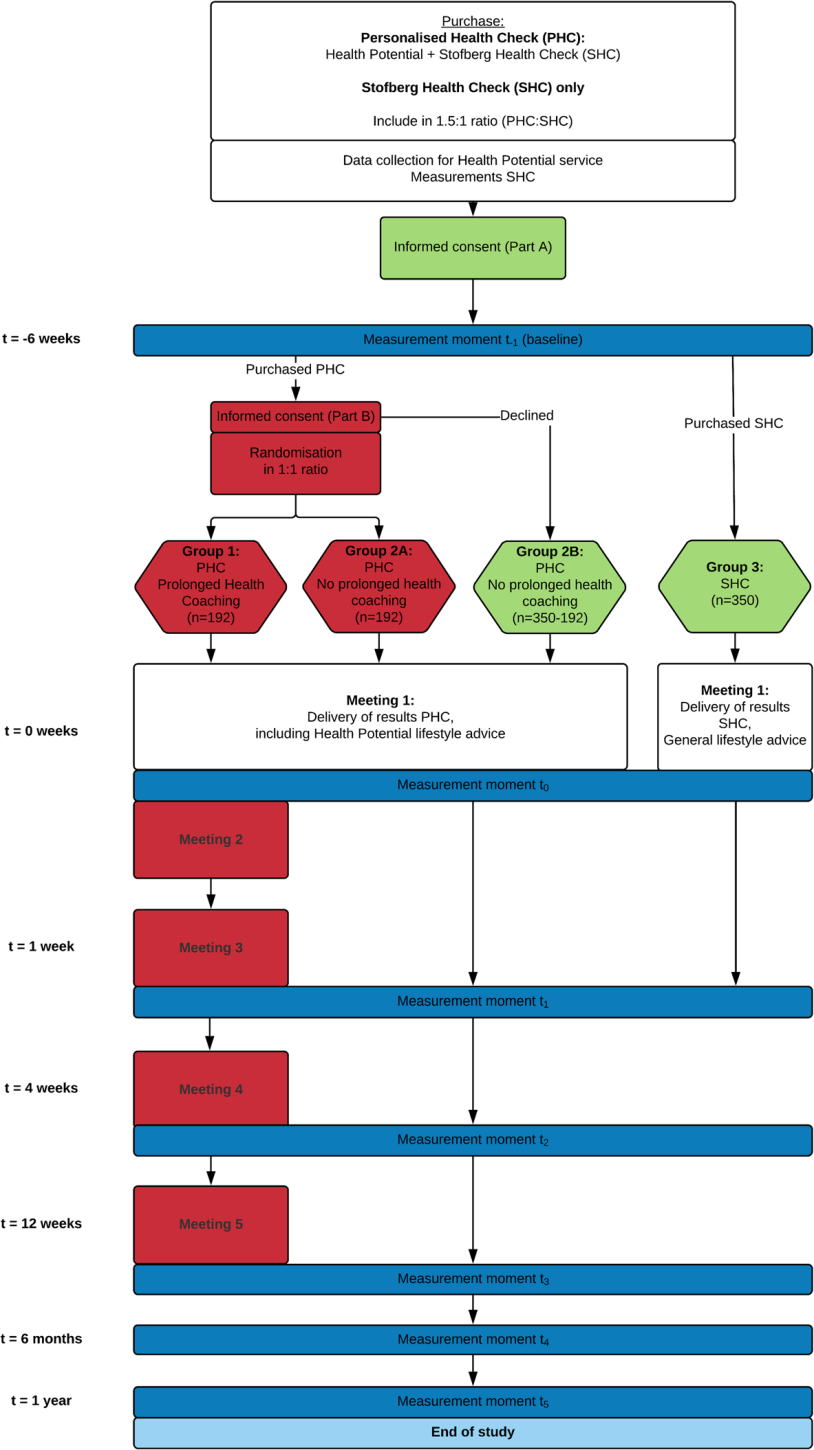
Conceptual framework

This study consists of two integrated trials. The first trial is a non-randomised two-armed controlled pre-test/post-test superiority trial (part A). Of one of those arms, participants can enter the second trial, which is a randomised controlled pre-test/post-test superiority trial (part B), on the condition that they consent to participate. In order to obtain sufficient participants for part B, participants in part A will be distributed in a 1.5:1 ratio. Participants will be randomised at a 1:1 ratio in part B. This results in three study arms (Fig. 1). However, due to eligible participants of part A potentially not consenting to participation in part B, study arm 2 can again be considered as two study arms: participants in arm 2a have consented to participate in part B whereas participants in 2b have not (see Fig. 1 for a visualisation).

#### Rationale for the study design

The study is designed in a way to reflect the ‘real world’ situation in which the participants are not randomised for part A and all participants pay a (reduced) fee for the services. The reason behind this approach is that we expect a certain level of ‘pre-motivation’ and possibly other different personal characteristics (e.g. personality, socio-economic status, income, level of education, gender, age) among clients of the Health Potential® service, particularly when an additional ‘barrier’ exists of a fee that has to be paid for the service.

Due to the nature and cost of the products, and consistent with Rogers’ theory of early adopters [21], the study population is likely to consist of (apparently) healthy individuals aged 18 and over, expected to have higher social status, higher financial resources, and higher education, and who are relatively health conscious.

### Study setting

Part A of the study takes place through online questionnaires, which participants can fill out at home. The additional lifestyle counselling will be offered through certified counsellors located in South-Limburg, The Netherlands.

### Participants and recruitment

Participants for part A of the study will be recruited from the regular customers at local prevention clinics who have purchased one of the following products:

- Product 1: *personalised health check* (PHC). The PHC consists of the Health Potential® service (future health) and a general health check of the customer’s current health.
- Product 2: a general health check of current health only (GHC).

Potential participants will be informed of the study by the local responsible investigator of each clinic. Eligible participants for part A are apparently healthy individuals aged 18 and over, with a Dutch language level similar to language proficiency level B2, not currently following any prescribed dietary or other guidelines, not pregnant or trying to become pregnant, authorised to make autonomous decisions, able to independently visit the counselling clinic, and have Internet access and an email address. Participants who purchased product 1 are eligible to take part in part B of the study. Enrolment will be open for 1 year.

### Sample size and power calculation

The sample size calculation is based on the proportion of participants who have changed their behaviour (being in the action/maintenance stage of behaviour change; see ‘Study parameters’) for at least three of the received advices at follow-up in the overall analysis. The type I error rate was set at 0.05 and an additional 20% was added to compensate for loss to follow-up in part A.

*Part A*: The smallest difference is expected between arms 2 and 3. It is estimated that with (291 + 20%=) 350 participants in each arm, part A has a power of 80% to detect a proportion of 0.3 in arm 2 versus a proportion of 0.2 in arm 3.

*Part B*: Study arm 1 will be compared to arm 2a. It is then estimated that with (160 + 20%=) 192 participants in each arm, part B has a power of 80% to detect a proportion of 0.45 in arm 1 versus a proportion of 0.3 in arm 2a. As in part A 548 participants will be included in the PHC condition, this will give a margin of (548 − 384=) 164 for participants not consenting to participate in part B. Together this results in a total number of participants of (192 + 350 + 350=) 892.

### Blinding, randomisation and treatment allocation

Participants who purchased the PHC and who consented to participate in part B will be electronically randomised to receive the additional lifestyle counselling. Randomisation will be stratified by local clinic, and block randomisation will be used with block sizes of 4 within each stratum. The health coach will subsequently implement the appropriate intervention.

Although blinding is not possible for the participants and the health counsellors delivering the intervention, the data analyses will be performed in a blinded format through preparation of the dataset by an independent researcher.

### Investigational behavioural intervention

Part A does not contain an intervention. In part B, the participant receives additional lifestyle counselling. The lifestyle counselling consists of four additional personal face-to-face sessions with a purpose-trained lifestyle counsellor. The counselling follows four phases matching four core questions [22] and is delivered using principles of motivational interviewing [23, 24]:

- Counselling phase 1: “Am I at an increased risk because of my lifestyle?”
- Counselling phase 2: “Which choices are suitable for me?”
- Counselling phase 3: “How do I translate my choices to behaviour?”
- Counselling phase 4: “How can I maintain my change in behaviour?”

All lifestyle counselling sessions are done by the same certified lifestyle counsellors for one participant. The sessions take place at *t* = 0 week, *t* = 1 week, *t* = 4 weeks and *t* = 12 weeks (see Fig. 1, sessions 2–5) and will last approximately 45–60 min per session.

### Study parameters

**Fig. 2:**
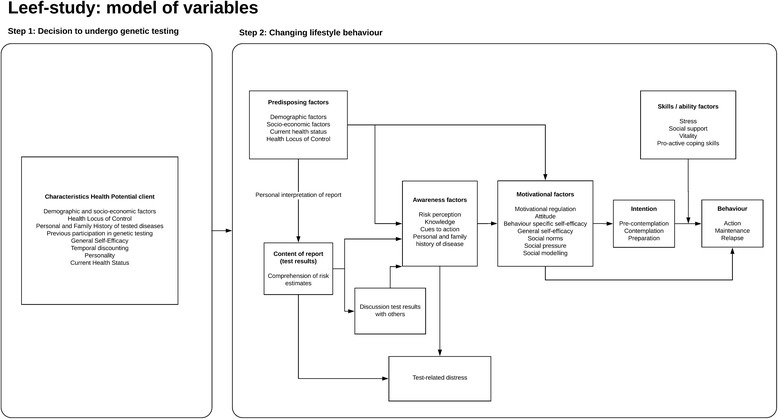
Flow chart of the study

To study the effect of Health Potential® on behaviour change, a conceptual framework has been developed (Fig. 2), based on a previously proposed framework for PGT by Bloss et al. [8] and the I-change model [25]. The framework consists of two steps that are relevant for eventual behaviour change: (1) purchasing Health Potential® and (2) behaviour change as a result of Health Potential®. Determinants included in both steps are based on the literature on PGT [20, 26–36] and the I-change model [37].

**Fig. 3:**
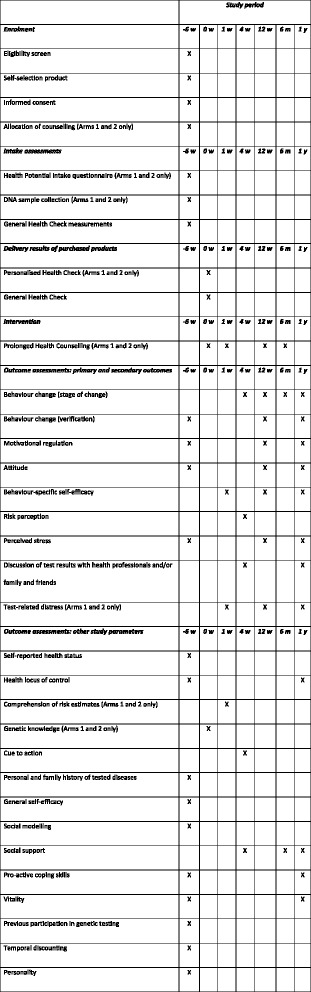
Schedule of enrolment, interventions and assessment

**Fig. 4:**
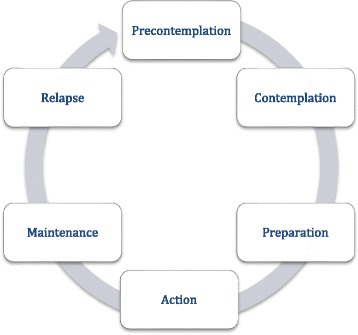
Transtheoretical model [43–45]

All parameters (Table 1) are measured using online questionnaires, as part of part A of the study, and are saved in a coded format. Measurements take place at *t* = −6 weeks (baseline), *t* = 0 week, *t* = 1 week, *t* = 4 weeks, *t* = 12 weeks, *t* = 6 months and *t* = 1 year (Figs. 3 and 4).

**Table 1:** Outcome parameters and other parameters

Outcome parameters	
Behaviour change (stage of change)	Questionnaire containing 1 question per advice received to determine the current stage of change (transtheoretical model (Fig. 4) [43–45]).
Behaviour change (verification)	Physical activity: the validated Dutch version of the International Physical Activity Questionnaire (IPAQ) [46]. Smoking: questions on current smoking behaviour and quantity of smoking. Dietary and alcohol intake: questions on regular intake of all food groups and alcoholic consumptions, for which advice is received by the participant. Body weight and waist circumference: measured at home by the participant, following detailed instructions and using the same weighing scale each time.
Motivational regulation	Validated Treatment Self-Regulation Questionnaire (TSRQ) [47], translated into Dutch. This questionnaire has been modified to ask motivational regulation with regard to the ‘advice given’ and ‘healthy lifestyle’.
Attitude	Questionnaire consisting of 7 Likert scale items on outcome expectations, 16 Likert scale items on the reasons for (not) undergoing testing by Health Potential®, 50 Likert scale items on the attitude towards five primary lifestyle behaviours, 1 Likert scale item on the importance of preventing disease, and 6 items on the evaluation of the Health Potential® report.
Behaviour-specific self-efficacy	Questionnaire which was developed using the ‘Guide for constructing self-efficacy scales’ by Bandura [48]. It includes one self-efficacy question for each advice given in which the participant indicates on a scale of 0–100% how sure they are of their ability to follow the advice.
Risk perception	Likert scale questions on the perceived probability of getting and severity [37] of each of the diseases included in the standard Health Potential® service.
Perceived stress	Dutch translation of the validated 14-item Perceived Stress Scale (PSS) [49].
Discussion of test results with health professionals and/or family and friends	Multiple-answer question in which the participant can indicate with whom the results were discussed.
Test-related distress (arms 1 and 2 only)	Dutch translation [50, 51] of the validated Impact of Event Scale (IES), which has been used previously to study test-related distress after genetic testing for common disease risk [39].
Other study parameters	
Self-reported health status	Dutch translation of the validated RAND 36-Item Short Form Health Survey, V2 (SF-36 V2) [52, 53].
Health locus of control	Dutch translation of the validated Multidimensional Health Locus of Control (MHLC) Scale [54, 55].
Comprehension of risk estimates (arms 1 and 2 only)	Questions based on the questions used by Kaufman et al. [56].
Genetic knowledge (arms 1 and 2 only)	Dutch translation of the questions as used by Carere et al. [57].
Cue to action	Two Likert scale questions on whether the participant felt the Health Potential® report and the general health check were a cue to action.
Personal and family history of tested diseases	Questionnaire asking about personal and family history.
General self-efficacy	Validated Dutch General Self-Efficacy Scale (DGSES) [58].
Social modelling, social support	One Likert scale question per advice received.
Social support	One Likert scale question per lifestyle domain (diet, alcohol, physical activity, smoking, body weight).
Pro-active coping skills	Validated Utrechtse Pro-actieve Coping Competenties (UPCC) questionnaire [59].
Vitality	Validated Vita-16 [60].
Previous participation in genetic testing	Two yes/no questions.
Temporal discounting	Validated 10-item Delaying Gratification Inventory short form, translated into Dutch (DGI-10) [61].
Personality	Validated Dutch IPIP-50 [62].

### Statistical analysis

Where appropriate, the analyses will be performed according to an intention-to-treat (ITT) approach (part B only) and per-protocol approach. Data will be analysed using STATA (StataCorp. Stata Statistical Software, College Station, TX: StataCorp LP). Statistical significance is assumed at a *p* value of 0.05, and no correction for multiple testing will be applied.

### Ethical considerations

Due to the nature of the researched population and the very limited risks associated with the study, no data safety monitoring board or safety committee is deemed necessary. No interim analyses will be performed.

The full protocols, for both parts A and B, can be found on https://dataverse.nl/dataset.xhtml?persistentId=hdl:10411/XKOM8H identifier 10411/XKOM8H (see also Additional file 1).

## Discussion

To our best knowledge, this is the first trial investigating such a wide range of determinants with regard to the effect of a disease prevention service based on both genetic and lifestyle components of disease risk. The study will contribute to the debate on whether genetic prevention services can be effective in improving lifestyle behaviour in certain customer groups and will help identify actionable determinants that contribute to an increased effect. Health Potential® will support the individual in making informed decisions about their health and lifestyle, which will become increasingly important in the current paradigm of personal responsibility for one’s health.

The study has several strengths and risks. Firstly, the pragmatic approach of the study will give a good indication of the effect in a real-life situation after implementation, resulting in a small theory-practice gap. However, on the one hand, it may also result in greater heterogeneity and thereby a dilution of the effect. On the other hand, the expected population of mostly early adopters might again reduce heterogeneity. Secondly, due to this pragmatic approach and the relatively high cost of genetic testing, the study population can be expected to be a selective group of higher educated individuals with a relatively high income. As health and lifestyle are associated with socio-economic position, services like Health Potential® may contribute to increasing health inequalities when the test is purchased personally. In the future, employers or municipalities may subsidise such tests for their employees or certain groups of inhabitants, which could diminish this effect. Finally, the outcomes include a wide range of actionable determinants of behaviour change, which will allow us to investigate through which determinants the effect on behaviour change is achieved. Knowing these explanatory variables may then inform us about the determinants that need to be addressed further in order to achieve a greater effect on behaviour change, direct recruitment strategies, and guide further development of genetic testing services. In addition, identification of specific groups of customers in which an effect is or is not seen will further help to tailor strategies.

PGT services, such as Health Potential®, also carry some risks and benefits for the consumer. Firstly, because of the high psychological impact of genetic testing for diseases with a strong genotype-phenotype interaction, it is under debate whether genetic testing for common disease risk also leads to too high levels of distress among participants. However, recent studies have suggested that the level of distress is minimal [7, 14, 38, 39]. The close contact between client and coach is hypothesised to further reduce any worry or distress and in case any serious distress arises, this can be recognised at an early stage and appropriate action can be taken. Whether this holds true will also be examined in this study. Secondly, a serious risk of PGT services is misinterpretation of test results by consumers [16]. Consequences of misinterpretation may be undermined motivation (e.g. interpreting a reduced risk as definitely not getting the disease), unnecessary worry and distress, and a burden on the health care system through requesting unnecessary medical tests and asking for help from health care professionals to interpret the results [7, 40]. To address the issue, the Health Potential® report has been based on literature on communication of disease risks [41] and the results are explained to the client face-to-face by a certified Health Potential® coach. Finally, Health Potential® has a strong connection with her end users, including both coaches and clients. In the development of the supporting software and the communication of results, end users have been involved. This involvement is crucial for the acceptance and therefore viability of a service [42].

This research can provide valuable insights into the effectiveness of PGT services. These findings can be used in the development and improvement of lifestyle interventions enriched with modern genetic advancements.

## Trial status

The trial is currently in preparation.

## Additional file

Additional file 1: SPIRIT 2013 checklist: recommended items to address in a clinical trial protocol and related documents. (DOC 123 kb)

## Abbreviations

DTC: Direct-to-consumer
GHC: General health check
METC: Medisch Ethische Toetsings Commissie (Medical Research Ethics Committee (MREC))
PGT: Personal genomic testing
PHC: Personal health check
WHO: World Health Organization
